# Perceptual response patterns in rhesus macaque attentional set shifting are associated with inflexible behavior

**DOI:** 10.3758/s13415-025-01374-1

**Published:** 2026-01-08

**Authors:** Colleen E. McGonigle, Steven W. Gonzales, Kathleen A. Grant, Christopher C. Lapish

**Affiliations:** 1 Department of Psychology, IUI, Indianapolis, IN USA; 2 Division of Neuroscience, ONPRC, Beaverton, OR USA; 3 Department of Anatomy, Cell Biology & Physiology, IUSM, 320 W. 15th Street, Neuroscience Research Building, 300C, Indianapolis, IN 46202 USA

**Keywords:** Animal models, Cognitive control, Decision-making

## Abstract

The attentional set shifting task has been used across species to assess cognitive flexibility. Each phase of the task requires an animal to update their behavior based on a different rule that determines which feature of a complex stimulus is signaling a correct response. The present study used male and female rhesus monkey data to assess how response patterns were used in attentional set shifting, as it relates to cognitive flexibility. The formation of an attentional set where trials to criteria were higher in the extradimensional shift than the intradimensional shift, as well as elevated trials to criteria at the first reversal phase were observed after experience with the task. Bayesian analysis was used to detect the emergence of dominant response patterns, reflecting consecutive trials on which monkeys consistently selected stimuli sharing a particular feature: spatial location, shape, or color. This analysis revealed that monkeys that successfully complete the task do not heavily rely on a single dominant response pattern until they have accumulated sufficient evidence about which exemplar signals reinforcement. On the other hand, monkeys that do not complete the full task tend to deploy stronger perceptual response patterns, either indicating a tendency to perseverate on response patterns that are not reinforced or a tendency to sequentially attempt perceptual response patterns. The current data suggest that higher cognitive flexibility involves response patterns that are more advanced than following single perceptual domains. Future work will focus on how the temporal evolution of response patterns are related to cognitive flexibility.

## Introduction

Cognitive flexibility is the ability to adapt behavior in response to a changing environment. Poor cognitive flexibility is a risk factor for multiple psychiatric disorders, including suicidality, posttraumatic stress disorder, and alcohol use disorder (Ben-Zion et al., 2018; Hausman et al., 2020; Nippert et al., 2024). To better understand this association between cognitive flexibility and psychiatric disorders, it is necessary to determine cross-species commonalities in cognitive processes to implement flexible decision-making. If points of contact can be identified, this would allow translational investigation of underlying mechanisms of flexibility (De Falco et al., 2021; Shnitko et al., 2019). Toward this goal, the current study examined the latent response patterns that guide flexible decision-making in nonhuman primates.

The Wisconsin Card Sorting Task is widely used in clinical tests of cognitive flexibility, using a shifting set of rules with a deck of playing cards (Berg, 1948). This task requires the trial-and-error generation of a hypothesized rule as well as modification in response to environmental feedback. The test is sensitive enough to discriminate between healthy participants and those with substance use disorders (Faustino et al., 2021). This task has been adapted in rodent and nonhuman primate models in the form of the attentional set shifting task (ASST) (Birrell & Brown, 2000; Heisler et al., 2015). Rodent research on cognitive flexibility has been invaluable for dissecting the role of the prefrontal cortex in set shifting (Birrell & Brown, 2000; Bissonette et al., 2013; Wang et al., 2019). Nonhuman primate research has allowed for longitudinal experimentation, linking cognitive flexibility as a risk factor for the avidity for alcohol (Shnitko et al., 2020). It is important to note that within- and between-animal sources of variance affect performance on the attentional set shifting task. Within-subject variance in well-trained rodents is observed in poor test–retest reliability, complicating the use of this task to measure a stable trait (Tait et al., 2017). Attentional mechanisms are known to be impacted by both state and trait anxiety and affect, suggesting that within-subject variability could result from either state or trait-dependent mechanisms independent of cognitive flexibility (Hur et al., 2015; Matthews & Zeidner, 2012; Pacheco-Unguetti et al., 2010).

Most analyses have focused on measuring cognitive flexibility by measuring how many attempts it takes an individual to set shift. Fewer studies have assessed how an individual searches for and implements a new rule through the trial-and-error process (Brown & Tait, 2016). The mechanisms underlying the different phases of the task are different, with the orbitofrontal cortex involved in reversal phases (McAlonan & Brown, 2003), and the medial prefrontal cortex supporting the extradimensional shift (EDS), for example (Birrell & Brown, 2000). Prefrontal cortex-dependent processes, such as the extradimensional shift of ASST, have been shown to be accomplished by “moments of insight” where sudden neural state changes accompany rapid abandonment or adoption of rules (Durstewitz et al., 2010). This rapid shifting behavior is observed more strongly with overtraining, suggesting an interaction with a learned model of the task (Bartolo & Averbeck, 2020; Dhawan et al., 2019). Moreover, nonhuman primate work with detailed Bayesian methods has shown that monkeys build a model of task structure as they gain experience and perform reversals specifically with an increasingly strong model-based approach (Costa et al., 2015; Jang et al., 2015). Due to these mechanisms, an analysis strategy that allows for presolution dissection is beneficial to understand the ways in which animals organize their behavior while searching for rules.

Choices in the ASST and more broadly tend to be balanced between exploration and exploitation, looking for new adaptive choices and making choices that have worked in the past (Caso & Cooper, 2022; Mehlhorn et al., 2015). Exploration is both a mechanism for collecting new information and a mechanism for ensuring that the selected option is the most beneficial choice. Tonic exploration, or the investigation of alternative options even when the task does not require it, is inversely related to perseverative behavior (Ebitz et al., 2019). Excessive exploitation can be linked to habitual behavior, including substance use, and is often associated with low cognitive flexibility (Corbit & Janak, 2016). Rodent ASSTs have demonstrated animals that perform poorly tend to rely upon task-irrelevant response patterns, such as selecting for the spatial location of a stimulus, to explore environmental contingencies (Wang et al., 2019).

The present study investigates the hypothesis that perseveration in perceptual response patterns, particularly of spatial patterns, is also demonstrated by nonhuman primates during the ASST and that this will be linked to lower cognitive flexibility. In the data analyzed herein, monkeys perform a self-paced version of ASST, meaning that they elect when to initiate a trial and how many trials to complete within the session. This results in different levels of task experience between animals and thereby limiting the ability to investigate individual phases, such as EDS in between-subject designs. Bayesian analysis allows for dissection of task approaches without requiring specific achievement within the task (Wang et al., 2019). This approach also allows for the assessment of multiple response patterns and their competition with one another (De Falco et al., 2021; Durstewitz et al., 2010). Our conclusions support that spatial response patterns dominate among poor performers, and subthreshold response patterns are closely linked to high performance.

## Materials and methods

Experimental data and procedures come from the Oregon National Primate Research Center under Dr. Kathleen Grant. All procedures were conducted according to the Guide for the Care and Use of Laboratory Animals (National Research Council of the National Academies, 2011) and approved by the Oregon National Primate Research Center Animal Care and Use Committee. Full methods associated with data collection and prior analysis can be found in Shnitko et al. (2017) and Grant et al. (2022).

### Animals

Young adult rhesus monkeys (MATRR cohorts 14, 16, 17, 18; *Macaca mulatta*; *n* = 48, 18 females) were obtained from the breeding colony of Oregon National Primate Research Center and were tested in four cohorts.

Monkeys were housed in social testing environments. Each was housed in a cage with a removable horizontal partition, which was removed for 1–2 h/day of paired housing conditions. Eight to 12 animals were housed and tested together at a time. The monkeys had a panel in their cages with a dowel, two drinking spouts, and a food receptacle (Med Associates Inc, United States). A computer-controlled LCD monitor (11 × 13.25 inches, Dell Inc., Model E1715S) with attached touch-sensitive screen overlay (Keytec Inc., Model OPTIR Touch PPMT, United States) was included in each panel. Programming used a National Instruments interface and LabView software (LabView 2011, SP1, National Instruments, Texas, United States). Through this set up, monkeys operantly performed cognitive testing, self-administered water and alcohol, and earned food. Monkeys were fed a diet of nutritionally complete 1 g of banana-flavored pellets (TestDiet, United States) and fresh fruit.

### Attentional set shifting

Monkeys performed the ASST using a touchscreen built into the wall of their housing cages. Through the task, these touchscreens display pairs of complex stimuli, and the monkeys are tasked with determining which stimulus is associated with delivery of 1 g of banana-flavored food pellets. All stimulus features are visual, and perceptual features include spatial location of presentation on the screen, shape, and color. Prior to testing, animals went through a photo preference test to assign a preferred image for use as a secondary reinforcer in this task. At the beginning of the task, each correct response is marked by the presentation of the preferred photo as well as a food pellet. Over the course of a 7-day training period, the fixed ratio for earning the banana pellet increases to 3, but the preferred photo is still presented each trial on an FR1 schedule. Incorrect trials result in a 10-s timeout. Animals have 30 s to respond to presented stimuli before an omission is recorded, and a new trial begins. Monkeys initiate the start of each trial by pressing on the touchscreen. Sessions can last for a maximum of 45 min each day but terminate upon successful completion of the final phase of the extradimensional reversal task phase.

The monkey ASST is self-paced, meaning that each animal chooses when to initiate a trial and how many trials to complete. It is valuable to note that task performance may not be solely impacted by cognitive flexibility in this self-paced paradigm, because environmental distractors and motivational differences may lead to both within- and between-subject variability. Criteria for progressing from one phase to the next is 12 of 15 running trials correct. Monkeys train for 7 days, during which they are acclimating to touchscreens and the task demands, sometimes with experimenter help. This training period represents a period where many monkeys made significant progress in task familiarization and performance, which is omitted from analysis due to the experimenter’s involvement during the training period that does not accompany the testing period. Following these initial 7 days, all monkeys progress to the predrinking test period consisting of 30 sessions. These 30 sessions are analyzed in the present study. There is no training or exclusion criteria for advancing from the seven training sessions to the 30 testing sessions.

### Analysis strategy

To parallel analysis strategies established in rodent data, monkey performance was first evaluated based on trials to criterion, particularly as it relates to the extradimensional shift. To this end, linear regression was applied to the group-averaged trials to criteria for each phase of the task to observe improvements from task experience. Trials to criteria slopes for each animal were also calculated to capture individual improvements from baseline performance with task familiarity.

Monkeys were separated into high- and low-performing groups. Task completers were identified when monkeys reached EDS for a minimum of three consecutive sessions. Approximately half of the animals were classified as task completers under these criteria, while many of the task noncompleters did not progress beyond the first three phases of ASST. To assess the effects of experience on EDS performance, one-way ANOVA was used to assess the differences between performance on each phase of the task, with particular interest in the difference between the intra- and extra-dimensional shift phases. The data aligned to EDS completion (Fig. 3) omits the extradimensional reversal (EDR) because monkeys do not necessarily complete EDR on the first or last session that they completed EDS. The present monkey data has been previously analyzed with a multifactorial performance index that captures overall performance through the ratio of trials ending in error to the total number of trials, the session duration, and the maximal phase the animal reached during the session. In these papers, the slope of performance index, or the improvement in task performance over training, was the summary metric for cognitive flexibility. While the present study did not use this metric of performance, our definition of task completers and noncompleters had 93.75% agreement with the categorization of high and low performers, respectively, from previous studies using performance index slope (Grant et al., 2022). In response to the high number of task noncompleters, further analysis focused on the ways in which animals were organizing their behavior within a session.

### Bayesian analysis

Bayesian inference allows for relative quantification of the degree to which present evidence is congruent with past evidence. This is applied here to assess how strongly an animal appears to be organizing their behavior according to a perceptual response pattern. Response patterns assessed include making choices based on the shape, the color, or the spatial location of the stimulus. This approach has been used for attentional set shifting analysis in several rodent papers (De Falco et al., 2021; Wang et al., 2019). Note that rigorous Bayesian analysis has been utilized in related, but distinct, applications to model dynamics of reversal learning (Costa et al., 2015; Jang et al., 2015). This work has revealed important information about the way in which monkeys form beliefs and efficiently switch behavior through model-based behavior.

The overall evidence for a response pattern present on a given trial is given by a Bayesian posterior. This evidence is assessed for each of the three hypothesized perceptual response patterns.

$${Posterior}_{i} (x)={likelihood}_{i}(x) \times {Prior}_{i}(x)$$

The likelihood value attributes the weight with which new evidence competes with the previous evidence, represented in the prior. Past applications of Bayes to attentional set shifting have assigned a likelihood values, such as 0.64 when a choice consistent with the hypothesized response pattern is made and 0.36 when the opposite is made, allowing for accumulation along an asymptotic function. The present study makes use of an adjusting likelihood function that follows a sigmoidal curve:

$${likelihood}_{i}(x)=0.5+\frac{0.5}{1+ {e}^{-(z-6)}}$$

where *i* represents the trial, *x* represents the hypothesized response pattern, and* z* represents the number of consecutive choices in agreement with that response pattern. These likelihood values are multiplied by the prior evidence for that response pattern, providing an updated trial-by-trial posterior evidence, or b-value. These posteriors were normalized across all possible response patterns, ensuring that they always sum to 1. The nonlinearity of the sigmoid function allows evidence to rapidly increase after a critical number of consecutive choices and then level off, limiting perturbations caused by erratic or random behavior. Importantly, the number of trials required for this likelihood function to cross the evidence threshold in an ideal model is higher than when using a static likelihood value (Figs. 4A and B). The sigmoidal function used reaches its upper bound at the minimum number of trials to pass criterion (*n* = 12), allowing for the potential detection of response patterns even in animals that complete the task as efficiently as possible, and has its midpoint between four to five choices. Figure 4 demonstrates the features of this sigmoidal likelihood function. The ideal observer model used herein contained 12 consecutive trials of generated data where the stimulus selected is entirely consistent with one response pattern and never consistent with an alternative response pattern. This ideal model avoids the ambiguity that real data displays on trials where the randomization of stimulus features results in evidence supporting multiple response patterns.

Extensive work connecting neural and behavioral data shows that there are “moments of insight” where remapping in the activity in PFC ensembles corresponds to the acquisition of a new rule (Bartolo & Averbeck, 2020; Durstewitz et al., 2010). Bayes theorem averages previous and current evidence together, fundamentally encouraging gradual updates to the posterior. To increase sensitivity to abrupt shifts in behavior, a likelihood function was utilized when choices inconsistent with the hypothesized response pattern were made, rather than utilizing a static value. The implementation of this decrement function increased the number of detected shifts in behavior, as indicated by the times that the Bayesian posteriors crossed the threshold, and it also prevented the posterior values from getting stuck at very high values after periods where monkeys perseverated on a single domain (Figs. 4E and F). When a monkey departs from a string of consistent choices, the likelihood value for that departure trial is replaced by the previous trial’s inverse. In this way, when a monkey has only chosen the same stimulus feature a few times, the likelihood decrement is minimal, but after many consistent choices, the decrement function will be near its maximal value.

Ground truth data were generated from a random selection of subjects to provide a dataset where response patterns were hardcoded into the data. This provided a more objective way to detect response patterns in animal behavior. In this experiment, data were generated according to specific response patterns, such that spatial, shape, and color response patterns lasting 6–12 trials each were coded. This simulated data differs from the ideal observer in that complex stimulus identities were maintained, allowing for the possibility of ambiguity where a trial may provide evidence for multiple perceptual response patterns. These data were analyzed through our Bayes framework to ensure that the approach was consistently detecting response patterns when present. This ground truth dataset was also used to test the effect of the likelihood decrement function. Lastly, this data were shuffled iterations to determine if the evidence threshold for response pattern classification was appropriate.

The evidence threshold for similar analyses has most commonly been set at 0.6 (De Falco et al., 2021; Wang et al., 2019). To determine whether 0.6 was sufficiently rigorous to detect relatively dominant response patterns, we conducted a bootstrapped shuffling procedure with ground truth and subject data in which the temporal relationship between trials was randomized. The shuffling process was run 100 times for each animal or each ground truth dataset and then Bayesian analysis was run on the shuffled data to produce a distribution of posteriors observed from randomized stimulus selection. Ground truth data were additionally shuffled iteratively with varying windows of temporal data preserved. Data were shuffled with bin sizes of 1, 6, and 12 to assess the point at which sufficient data with known response patterns would result in Bayesian detection of a response pattern emerging.

GraphPad Prism (10.6.1) and MATLAB (R2022b) were used for data handling and analysis. Bonferroni post-hoc analysis for Chi-Square comparisons of response pattern proportions was conducted in R using Daniel Ebbert’s Github implementation (Beasley & Schumacker, 1995; Ebbert, 2019).

## Results

**Fig. 1:**
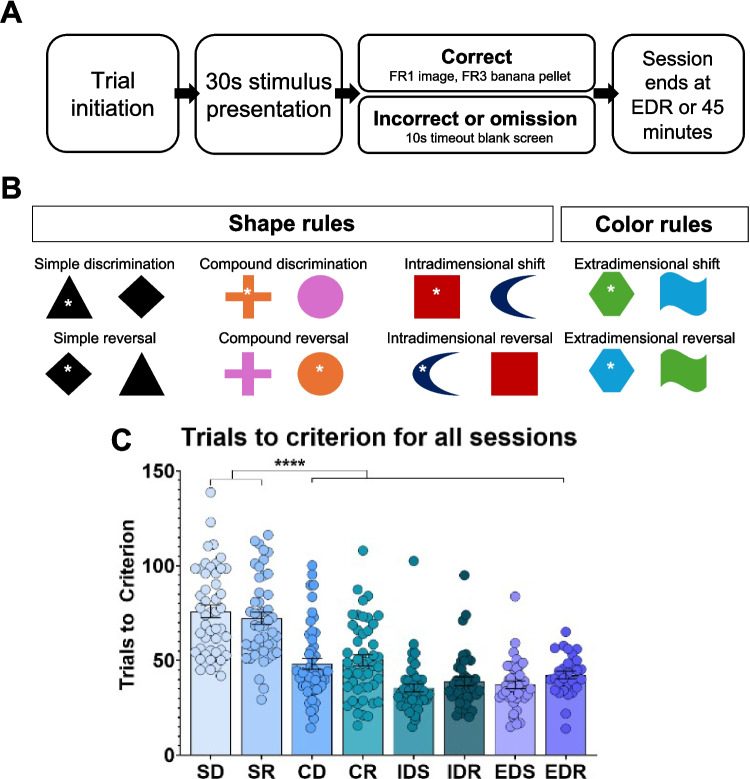
Experimental overview and performance metrics for each phase of the ASST. **A.** Attentional set shifting session overview. **B.** Example complex stimuli and reinforcement rules for each phase of the task. **C.** When trials to criterion are averaged across all sessions of training, there is a main effect of phase, and the initial two phases are significantly higher than all other phases (*p < 0.0001)

When data from all monkeys are collapsed across sessions and evaluated by trials to criteria, simple discrimination (SD) and simple reversal (SR) are higher than all other phases (Fig. 1C; one-way ANOVA F(7, 337) = 6.254, *p* < 0.0001).

**Fig. 2:**
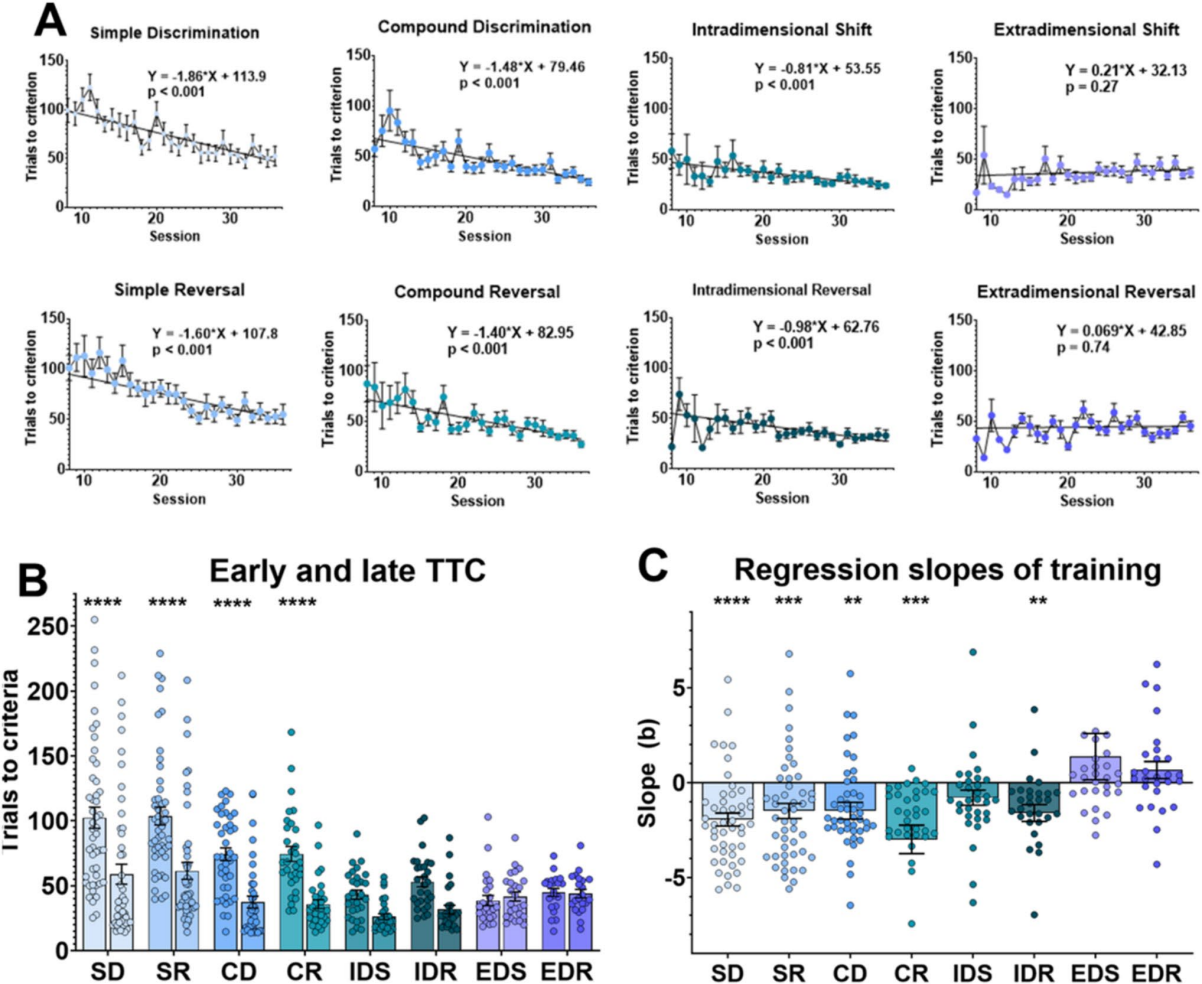
Experience improves task performance in all phases except the extradimensional shift and reversal. **A.** Performance, as measured by trials to criterion, improves with task experience in all phases except extradimensional shift and reversal. **B.** The change between the first three sessions (left bars) and the final three sessions (right bars) with each phase is significantly different for the first four phases of the task. **C.** Regressions on trials to criterion for individual animals were run for each phase and this also reveals a significant negative slope (indicating improvement) for all phases but IDS, EDS, and EDR (****p < 0.0001; ***p < 0.001; **p < 0.01)

To assess the effect of experience on performance, linear regression was performed on trials to criterion in each phase of the task across sessions (Fig. 2). Negative performance slopes are observed in the first six phases but not in EDS or EDR when all animals are averaged together in each session (Fig. 2A). Simple linear regression demonstrated significant negative slopes for all phases (b < − 0.81, F(1,455–1,512) > 30.97, *p* < 0.001) except for the EDS (b = 0.21, F(1,402) = 1.225, *p* = 0.27) and EDR (b = 0.069, F(1, 322) = 0.1091, *p* = 0.74). Because of the self-paced nature of the ASST, the data were next aligned to the first and last times that each animal completed each phase of the task. When the averages of the first three and final three sessions are compared for each animal, significant differences are observed for all phases up to intradimensional shift (IDS) (Fig. 2B). Two-way ANOVA revealed main effects of phase and timepoint, as well as an interaction between the two (interaction F(7, 260) = 4.594, *p* < 0.0001). Bonferroni multiple comparisons revealed significant differences for SD, SR, compound discrimination, and compound reversal (*p* < 0.0001), but not for IDS, intradimensional reversal (IDR), EDR, or EDS (*p* > 0.14). Finally, when performance slopes are calculated for individual animals across all sessions they performed, one sample *t*-tests revealed significant differences from zero for all phases (Fig. 2C: t(29–47) > 3.358, *p* < 0.0016) except IDS (t(31) = 2.001, *p* = 0.054), EDS (t(27) = 1.128, *p* = 0.27), and EDR (t(26) = 1.491, *p* = 0.148). Together, these analyses demonstrate that experience results in performance improvements for all phases of ASST except for the extradimensional shift and reversal phases, suggesting possible differences in mechanisms underlying these phases.

**Fig. 3:**
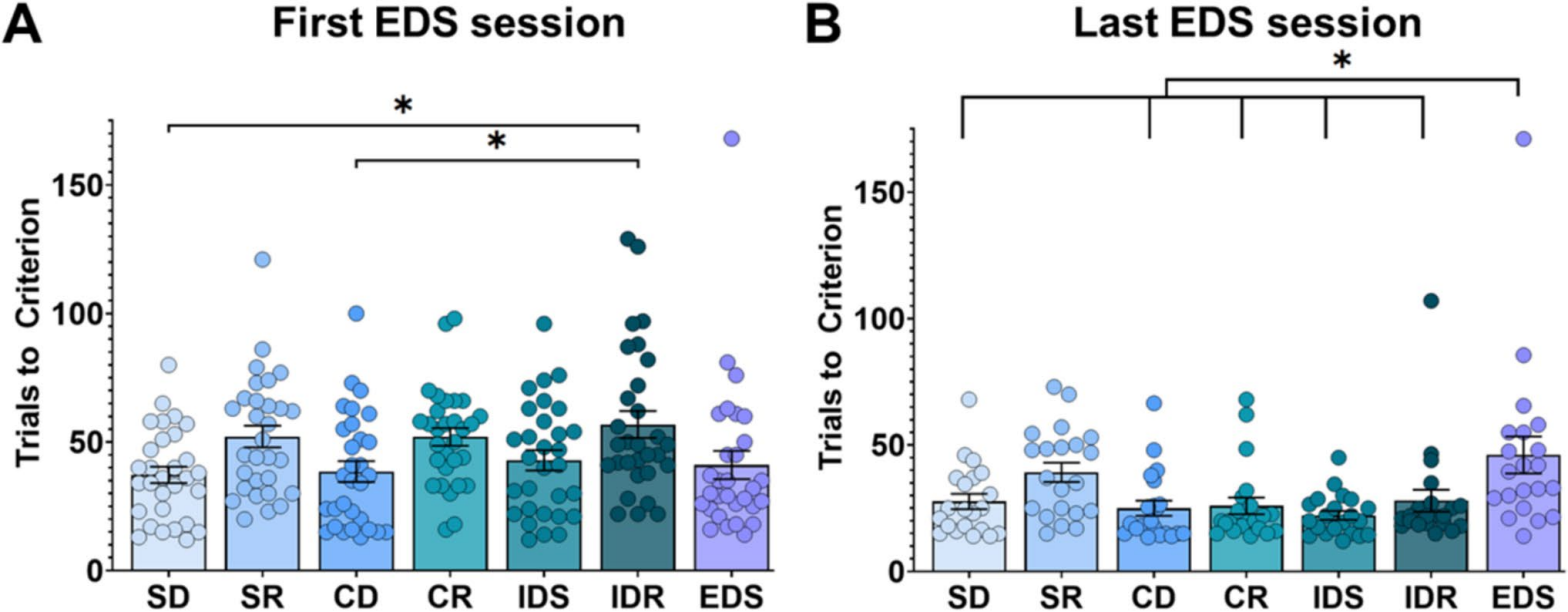
Experience is necessary for monkey performance to demonstrate attentional set formation. **A.** Trials to criteria differ across phases in early training, an effect driven by differences between the intradimensional reversal phase and SD and compound discrimination. **B.** By the end of the training period, performance is distributed more similarly to the typical pattern observed in rodents where EDS is elevated above the majority of the other phases. (*p < 0.05)

To first assess the monkey ASST through the typical rodent analysis approach, the distribution of trials to criterion across phases was analyzed. The expected pattern of trials to criterion from rodent literature is characterized by increases in the first of the reversal phases and on the extradimensional shift. Since rodent ASST data is typically analyzed the first time they complete the task, even in protocols where they go on to repeat the test, the monkey data were initially aligned to the first session in which each animal completed the extradimensional shift. In this first session, one-way ANOVA revealed a significant effect of phase (Fig. 3A: F(6, 203) = 3.224, *p* = 0.0048). Tukey’s multiple comparisons revealed that SD and IDR differed significantly (*p* = 0.0244) as well as compound discrimination and IDR (*p* = 0.0456). When the final session including EDS was analyzed, there remained a main effect of phase (Fig. 3B: F(6, 140) = 4.442, *p* = 0.0004). At this point, Tukey’s multiple comparisons revealed that EDS significantly differed from all phases (all *p* < 0.037) except SR (*p* = 0.90). Overall, these data demonstrate that monkey ASST data is most comparable to rodent ASST in experienced task-completing monkeys, who likely have developed a clear model of the task. Because these analyses were explicitly examining sessions where EDS was completed, only task completing monkeys were analyzed.

**Fig. 4:**
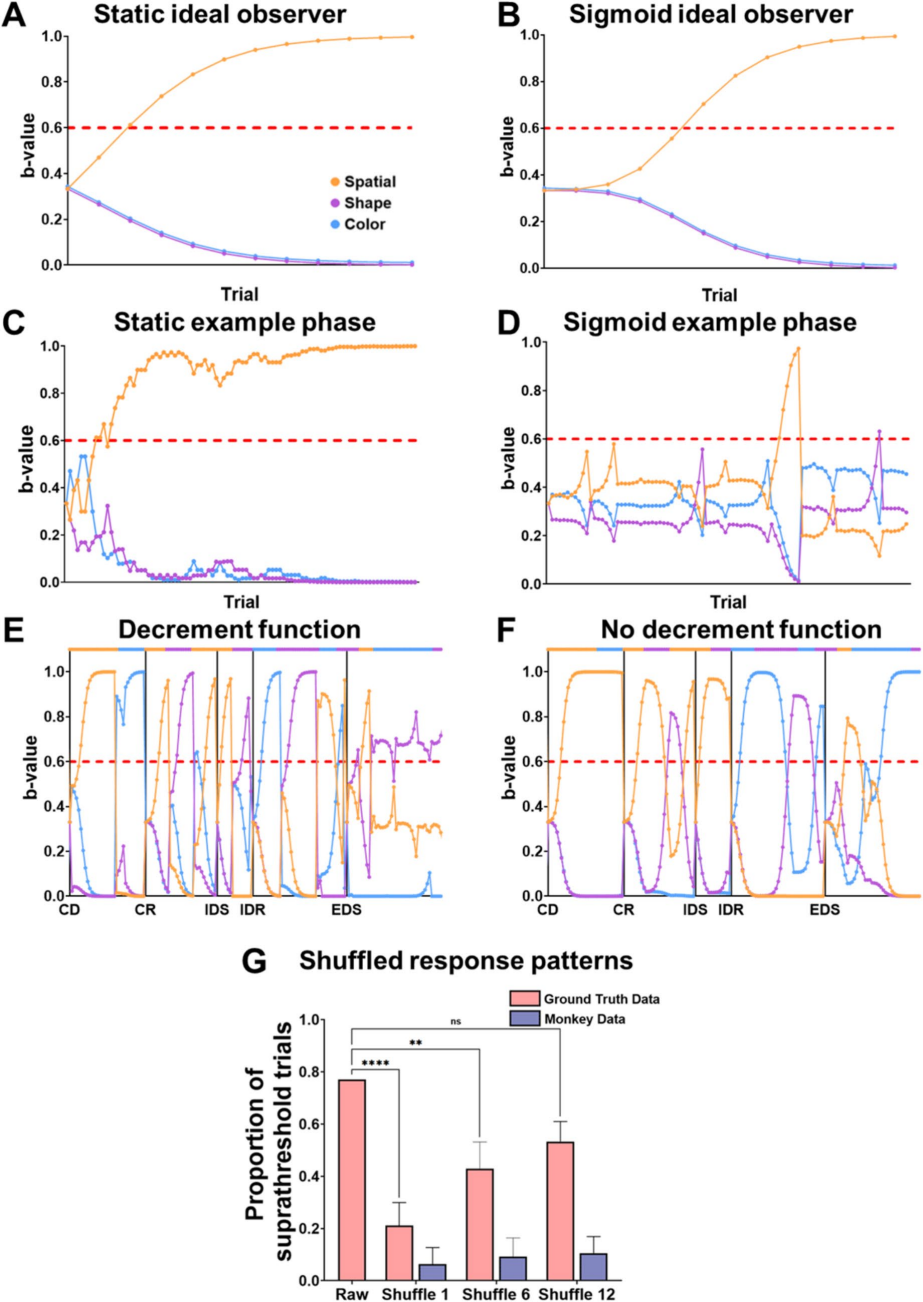
The inclusion of a sigmoidal likelihood function with converse decrement function improves the detection of nuanced response patterns with evidence threshold of 0.6. **A.** Static likelihood accumulation in ideal observer model. **B.** Sigmoidal likelihood accumulation in ideal observer model. **C.** Example phase where static likelihood results in detection of a single response pattern. **D.** Example of the improvement in nuanced response pattern detection with the inclusion of the sigmoidal likelihood. **E.** Ground truth data example with the decrement function included, featuring hardcoded response patterns lasting 6–12 trials and corresponding detection indicated by b-value passing threshold of 0.6. **F.** The same ground truth data without the inclusion of the decrement function causes more gradual shifts between response patterns. **G.** Iterative shuffling procedure demonstrating that when data with hardcoded response patterns has temporal relationship disrupted, response patterns are not detected but rather require temporal dependencies seen in behavior

Bayesian inference was employed to assess the consistency of behavior with hypothesized response patterns based on a monkey following a particular stimulus feature (spatial location, shape, color, or none). Likelihood values increased upward with each consecutive choice along a sigmoidal function, peaking at 12 trials to allow for detection of dominant response patterns even in monkeys completing a phase with the minimum required trials (Figs. 4A and B). Compared with static likelihood function that has been used in similar analyses, this sigmoidal approach improves the detection of nuanced response patterns (Figs. 4C and D). These likelihood that values were multiplied by the prior evidence for that response pattern, providing an updated trial-by-trial posterior evidence, or b-value. These posteriors were normalized across all possible response patterns, ensuring that they always sum to 1. Prior literature has used 0.6 as the threshold b-value at which a hypothesized response pattern is identified as “present” (Wang et al., 2019; De Falco et al., 2021). At a minimum, two response patterns are placed in competition with one another. It is critical to note that this approach detects relative dominance of response patterns rather than capturing absolute evidence for a response pattern. We make no explicit claim that the animals are thinking about specific strategies or rules, rather we attempt to detect behavioral patterns. Sensitivity to abrupt changes in behavior was enhanced with a decrement function, wherein a trial that was not consistent with a given response pattern was assigned the inverse of the previous trial’s likelihood value (examples shown in Figs. 4E and F). A shuffling procedure was employed to generate randomized data and Bayesian analysis was performed to ensure that the 0.6 threshold exceeded the range of possible values that could result from noise in the data (Fig. 4G). Two-way ANOVA revealed main effects of data source (ground truth vs. monkey: F(1,596) = 1437, *p* < 0.0001) and shuffling bin (F(3,596) = 122.4, *p* < 0.0001). Tukey’s multiple comparisons revealed that all groups significantly differed except the raw ground truth data from the 12-trial shuffle group. Only with large portions (12 trials) of “real” ground truth data, and not with monkey data, does the posterior significantly cross the 0.6 threshold when response patterns are known to be present, supporting the conclusion that threshold crossings do not occur as a result of random behavior.

**Fig. 5:**
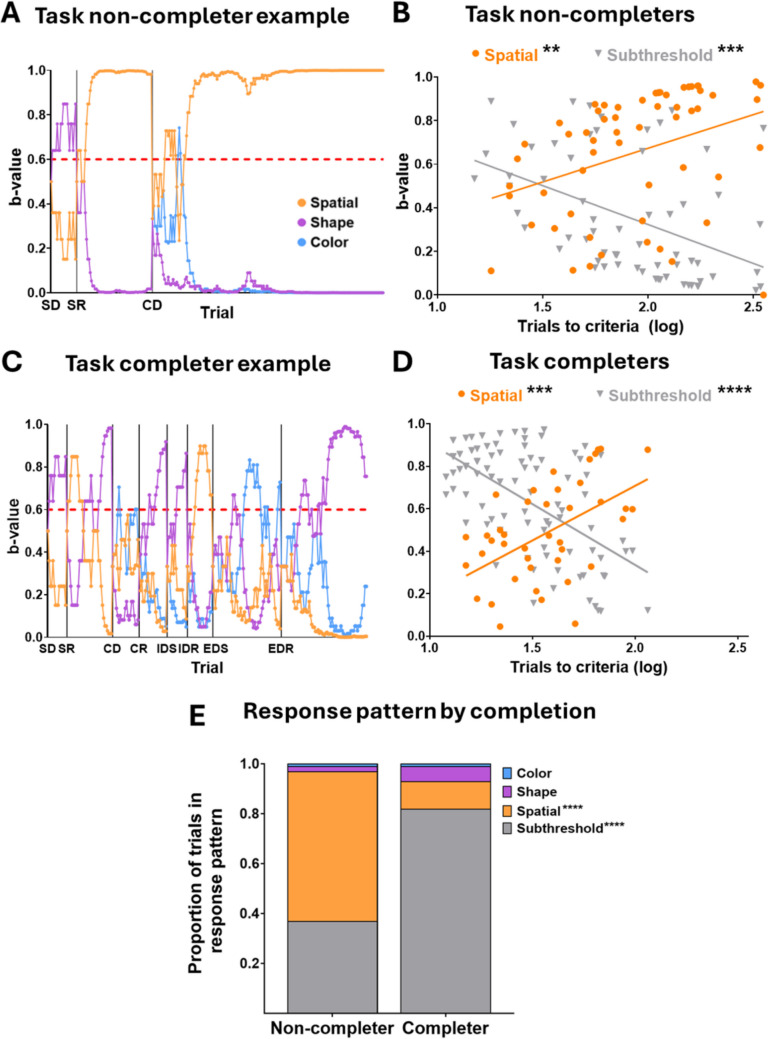
Bayesian analysis reveals differences in engagement with spatial and subthreshold response patterns between task noncompleters and completers. **A.** Example b-values for a task noncompleter session. **B.** Correlation for task noncompleters between Bayesian posteriors and trials to criterion for spatial and subthreshold response utilization. **C.** Example b-values for a task completer session. **D.** Correlation for task completers between Bayesian posteriors and trials to criterion for spatial and subthreshold response utilization. **E.** Proportion of trials spent in each response category for task noncompleters and completers (**p < 0.01; ***p < 0.001; ****p < 0.0001)

Example outputs from the Bayesian analysis are shown for a task noncompleter (Fig. 5A) and a task completer (Fig. 5C), visually demonstrating the reliance on a spatial response pattern in the task noncompleter and the duration of time spent below threshold in the task completer. Linear regression was performed to assess the correlation between the proportion of trials spent in spatial and subthreshold response categories and the trials to criterion, revealing positive correlations between performance and subthreshold patterns and negative correlations between performance and spatial response patterns. Trials to criterion were log transformed to address a left skew in the performance distribution (Fig. 5B: Task noncompleter spatial b = 0.31, F(1,56) = 8.225, *p* = 0.0058, subthreshold b = − 0.36, F(1,62) = 15.36, *p* = 0.0002; Fig. 5D: Task completer spatial b = 0.51, F(1,41) = 16.002, *p* = 0.0003, subthreshold b = − 0.57, F(1,92) = 49.45, *p* < 0.0001). Note for all these correlations, values of 1 and 0 were omitted from analysis to capture the balance between response patterns rather than sessions where an animal exclusively displays one response pattern. All phases were collapsed together, as the pattern of correlation was the same regardless of phase. These analyses revealed that regardless of performance level, relying on a spatial response pattern was positively associated with trials to criteria, indicating poorer performance with increasing adherence to a spatial response pattern. Conversely, the lack of reliance upon a perceptual response pattern, as indicated by the proportion of subthreshold response patterns, was negatively associated with trials to criteria, indicating better performance with decreased reliance on perceptual response patterns.

The response pattern distribution for task completing and noncompleting monkeys captures this effect. Pearson’s chi-squared test of independence reveals a significant difference between the response pattern proportions for task completers and noncompleters (Fig. 5E; χ^2^(3) = 52.83, *p* < 0.0001). Bonferroni post-hoc analysis (v0.1.2; Ebbert, 2019) revealed significant differences between subthreshold (*p* < 0.0001) and spatial response pattern (*p* < 0.0001) categories.

**Fig. 6:**
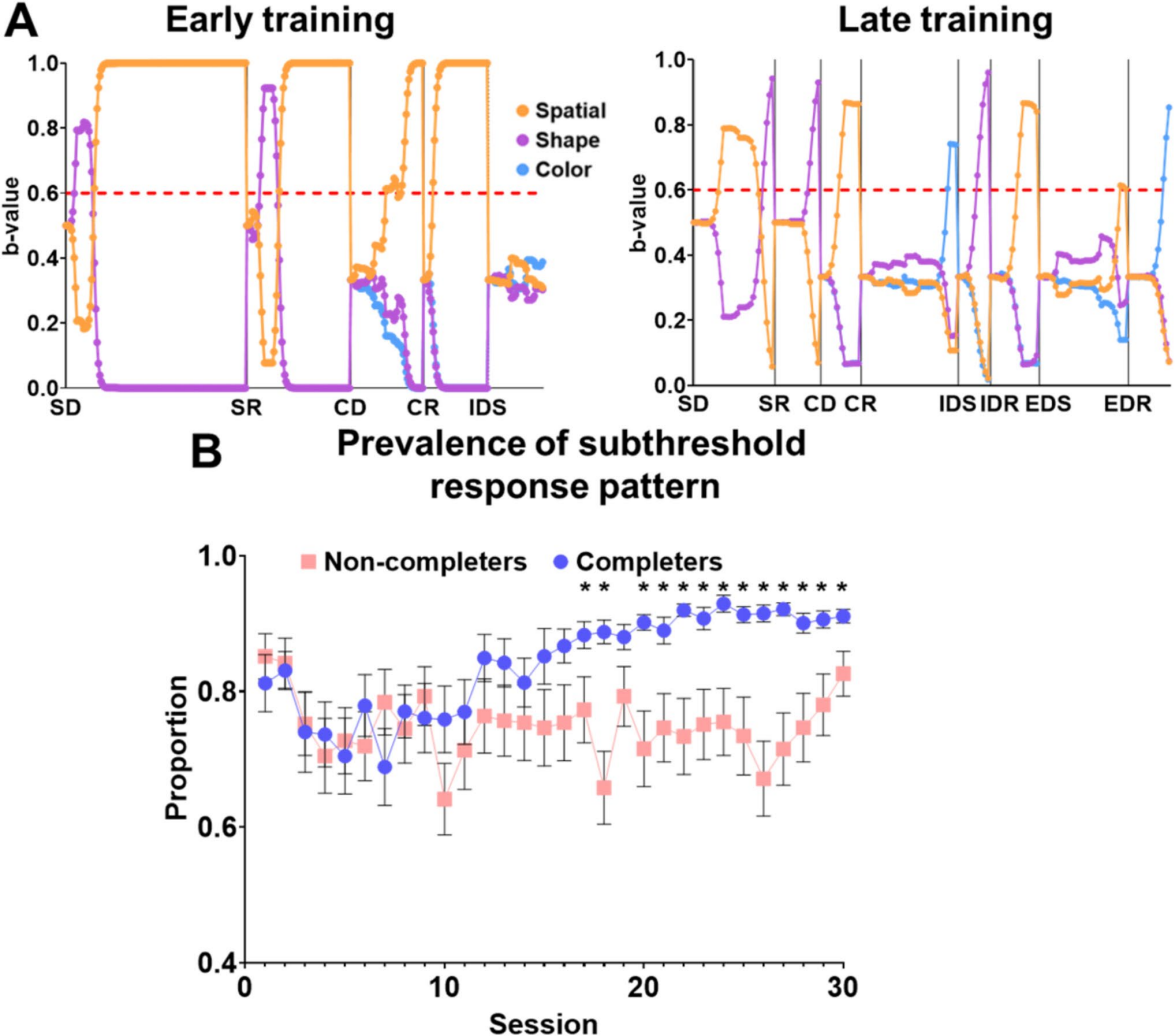
Perceptual response patterns emerge with experience. **A.** Example sessions for early (left) and late (right) training demonstrate initial preference for spatial response patterns, with a more nuanced behavioral pattern emerging with experience. **B.** The emergence of subthreshold dominance in task completers and noncompleters across sessions is significantly different, driven by significant differences after session 16. (*p < 0.05)

Differences in response patterns emerge with experience (Fig. 6A). When the proportion of subthreshold trials is compared session-by-session across task completers and noncompleters, two-way ANOVA shows main effects of session performance category, and a significant interaction between the two (Fig. 6B: F(29,1334) = 2.637, *p* < 0.0001). Fisher’s LSD multiple comparisons revealed significant differences on sessions 17, 18, and sessions 19–30 (t(24.24–30.43) = 2.082–4.313, *p’s* < 0.0458). All animals begin training with high levels of subthreshold response patterns, but the task completers recover this phenotype in late training. This suggests that the subthreshold category may be capturing two phenotypes: one that may include random exploratory behavior in early task experience, and one that is defined by refined responses in late task experience that are not driven by strong perceptual response patterns.

## Discussion

The major goals of this study were twofold. First, we assessed monkey set-shifting performance through an analysis approach typically used in rodent tasks to identify potential points of contact. We found that the repeated nature of the task in monkeys tends to yield improvements in task performance and bring performance in line with that observed in rodents. There are clear exceptions to this, however. Second, we assessed latent perceptual response patterns that guide decision-making in a monkey attentional set-shifting task. We observed that, as in rodents, reliance on a spatial response pattern is associated with poor performance. In addition, better performance in monkeys was associated with no detectable response pattern with the current Bayesian methods.

Performance in the early phases of the ASST tends to improve with experience. When all sessions are averaged together, we see that the simple discrimination and simple reversal phases have higher numbers of trials to criterion (Fig. 1). This is driven by the fact that some monkeys fail to make it past the first several phases of the task. Over the course of the experiment, monkeys, on average, demonstrate task improvement in every phase leading up to the extradimensional shift (Fig. 2). While approximately 50% of the monkeys are classified as task noncompleters and do not successfully reach the extradimensional shift, this phase improvement is still observed in approximately 75% of all monkeys. The extradimensional shift and reversal phases appear to be resistant to performance improvements with repeated exposure.

Because the monkey ASST involves repeated encounters with each set of rules, we were interested in seeing how performance compares with rodent performance in a common variation of the task where a single test session is employed. Early in training we do not see evidence that the monkeys have formed an attentional set, evidenced by the lack of a significant difference between IDS and EDS performance (Birrell & Brown, 2000). Rather, task familiarity is required for the monkeys to form an attentional set, producing the expected performance distribution (Fig. 3).

Bayesian analysis assessed perceptual response patterns used to solve the ASST (Figs. 4 and 5). This analysis revealed differences in response pattern utilization between task completing and noncompleting monkeys, consistently reflected in spatial response pattern dominance task noncompleters and subthreshold dominance for task completers. Many monkeys begin with high spatial response pattern, but task completers develop a lower level of suprathreshold response patterns as they progress through training (Fig. 6).

An initial focus of this project was to identify potential points of contact in the rodent and primate ASST. The testing environment differs in critical ways that likely impacts attentional allocation and could explain observed differences in performance. Rodents are tested in isolation, while monkeys are tested as a group. Therefore, monkeys have a greater range of distractors present in their testing environment. Rodents are also typically trained to a specific criterion prior to the first test, which can result in a form of overtraining (e.g. Bussey et al., 1997; reviewed in Tait et al., 2014). The task performance for the two groups of animals differs in several important ways also, but the most significant is that rodents consistently form an attentional set in their first encounter with the task, while monkeys only demonstrate an attentional set after repeated sessions. Rodent attentional set shifting can be performed multiple times with the attentional set remaining intact and improvements impacting phases of the task equally (Moench et al., 2020; Tait et al., 2017; Wallace et al., 2014). It was surprising that monkeys require repeated task experience to demonstrate the typical pattern of performance over phases as seen in rodents. Specifically in reference to where the simple reversal and the extradimensional shift tend to be elevated above other phases in trials to criterion.

Monkeys do not improve on the extradimensional shift with experience but do improve in all other phases (Figs. 2 and 3). We speculate that this may be attributable role of the prefrontal cortex in the extradimensional shift and that incremental learning does not benefit performance of the EDS as it does the other phases (Birrell & Brown, 2000; Floresco et al., 2008; Ragozzino, 2007). Lesions of the mPFC uniquely impair performance on the extradimensional shift (Birrell & Brown, 2000), and its key role may be to facilitate learning about previously redundant or irrelevant cues (Knott et al., 2024). The orbitofrontal cortex is critical for biasing attention away from irrelevant information through rule formation and maintenance, which requires mPFC involvement to overcome in the extradimensional shift (Sleezer et al., 2016; Ebitz et al., 2020). Together, these data highlight that the unique role of the mPFC in extradimensional shifting may drive our observation that the extradimensional shift does not improve with experience.

Furthermore, monkeys that reach the extradimensional shift may already be performing at near-optimal performance, thus limiting the ability to express improvement in performance with repeated sessions. The elevated trials to criterion observed in simple discrimination is more likely related to environmental distractors that slow engagement with the initial task phases. Since each testing session begins with the simple discrimination phase of the task, this model does not explicitly encourage monkeys to reach the end of the task and instead allows for each monkey to complete as much of the task as they are able and motivated to complete on a given day. Many other implementations of attentional set shifting resume testing each day with the maximum phase the animal reached on the previous day, facilitating their progression to the end of the task (Colacicco et al., 2002; Heisler et al., 2015).

Using trials to criterion does not capture important metrics of task performance. While many monkeys complete similar numbers of trials each day, those trials are distributed across different numbers of phases in each session. Task completers move through a larger number of phases and relatedly have lower trials to criterion on each phase. This results in skewed trials to criterion distributions because task noncompleters spend a disproportionately high number of trials in the early phases of the task. Other metrics have been developed to capture the finer points of performance that are not so strongly related to trials to criterion (Shnitko et al., 2017). Another approach has been to examine the presolution period or what the monkeys are doing in the trials prior to finding the reinforced rule in a given phase. This has most commonly taken the form of Bayesian analysis of response patterns (De Falco et al., 2021; Wang et al., 2019), but reinforcement learning models have also been used to probe the presence of behavioral strategies in these tasks (Bähner et al., 2025; Rothenhoefer et al., 2017; Taswell et al., 2021).

The response patterns that were examined here are perceptual in nature. It is likely that more advanced cognitive strategies are utilized by monkeys in this task. Our initial interest was in whether the monkeys were directing their attention preferentially toward specific stimulus features, but the dominance of the subthreshold response pattern category suggests that there are likely strategies guiding decision making that are not purely perceptual as we have defined it here. Other studies have probed the presence of alternation, or win-stay/lose-shift response patterns, but there are likely more possibilities that could be probed in the future (De Falco et al., 2021; Wang et al., 2019). The dominance of spatial response patterns in task noncompleters may also be capturing several phenotypes. This could represent both the “easiest” way to complete the task, because the monkey does not need to actively monitor the stimuli or engage in working memory processes to select a stimulus, and the most ecologically relevant strategy to default to when searching for a rule. Rodents and monkeys will tend to pay attention to spatial cues to guide foraging behavior, using spatial information may reflect an innate tendency of the animal (Griesius et al., 2023).

One major limitation of this study lies in the inability to probe the first time a phase is encountered across animals. For example, the standard assessment of an attentional set is to assess EDS as it compares to IDS, but this analysis is typically done when the animal has the same amount of experience with those two phases. Early analysis attempts focused on the initial encounter with a phase to align the analysis with rodent task design, where animals may only encounter each phase a single time. Because the task is self-paced, many monkeys reach the more advanced stages of the task during the initial training sessions, when researchers are still present in the room and the monkeys have not yet progressed to the FR3 reinforcement schedule. Relatedly, at any timepoint each monkey has a different history of task experience, which hinders the ability to collapse across groups, as is done in rodent studies where the task history is more controlled. A trial-by-trial analysis approach such as the Bayesian approach is helpful in this instance, because it facilitates the examination of individual data rather than placing the emphasis on group data.

Future work will explore subthreshold periods of behavior. It is possible that these periods, because they are observed more frequently in better performers, may represent the emergence of cognitive, rather than perceptual strategies. It could also represent either model-free exploration as an efficient way of searching for new rules following a phase shift or a phenotype of monitoring multiple hypothesized rules at once. In addition, previous work has explicitly connected cognitive flexibility to risk for future alcohol consumption (De Falco et al., 2021; Rodberg & Vazey, 2022; Shnitko et al., 2019; Lewis & Nixon, 2013). Future work will probe the relationship between cognitive flexibility and alcohol consumption in these subjects and address sex differences in this behavior.

## Data Availability

Data for the experiments is contained in the Monkey Alcohol Tissue Research Resource (MATTR) and is available upon request. The Bayesian analysis code can be found at https://github.com/cmcgoni/BayesianAnalysisMonkeyASST. Software utilized: MATLAB Version 9.13.0 (R2022b) RRID:SCR_001622; GraphPad Prism Version 10.4.1 RRID:SCR_002798.
